# Molecular and functional characterization of a conserved odorant receptor from *Aedes albopictus*

**DOI:** 10.1186/s13071-022-05158-1

**Published:** 2022-01-31

**Authors:** Ru Yan, Zhanyi Xu, Jiali Qian, Qiaoling Zhou, Huiming Wu, Yahui Liu, Yirong Guo, Guonian Zhu, Mengli Chen

**Affiliations:** 1 Institute of Pesticide and Environmental Toxicology, College of Agriculture and Biotechnology, Zhejiang University, Hangzhou, China; 2 The Key Laboratory for Quality Improvement of Agricultural Products of Zhejiang Province, College of Advanced Agricultural Sciences, Zhejiang A&F University, Hangzhou, China

**Keywords:** *Aedes albopictus*, Odorant receptor, (+)-Fenchone, Repellency, RNAi

## Abstract

**Background:**

The Asian tiger mosquito *Aedes albopictus* is a competent vector of several viral arboviruses including yellow fever, dengue fever, and chikungunya. Several vital mosquito behaviors (e.g., feeding, host-seeking, mating, and oviposition) are primarily dependent on the olfactory system for semiochemicals detection and discrimination. However, the limited number of studies hampers our understanding of the relationships between the *Ae. albopictus* olfactory system and the complex chemical world.

**Methods:**

We performed RT-qPCR assay on antennae of *Ae. albopictus* mosquitoes of different sexes, ages and physiological states, and found odorant receptor 11 (AalbOr11) enriched in non-blood-fed female mosquitoes. Then, we examined the odorant preference with a panel of physiologically and behaviorally relevant odorants in *Xenopus* oocytes.

**Results:**

The results indicated that AalbOr11 could be activated by ten aromatics, seven terpenes, six heterocyclics, and three alcohols. Furthermore, using post-RNA interference (RNAi) hand-in-cage assay, we found that reducing the transcript level of AalbOr11 affected the repellency activity mediated by (+)-fenchone at a lower concentration (0.01% v/v).

**Conclusions:**

Using in vitro functional characterization, we found that AalbOr11 was a broadly tuned receptor. Moreover, we found that AalbOr11 shared a conserved odorant reception profile with homologous *Anopheles gambiae* Or11. In addition, RNAi and bioassay suggested that AablOr11 might be one of the receptors mediating (+)-fenchone repellency activity. Our study attempted to link odor-induced behaviors to odorant reception and may lay the foundation for identifying active semiochemicals for monitoring or controlling mosquito populations.

**Graphical Abstract:**

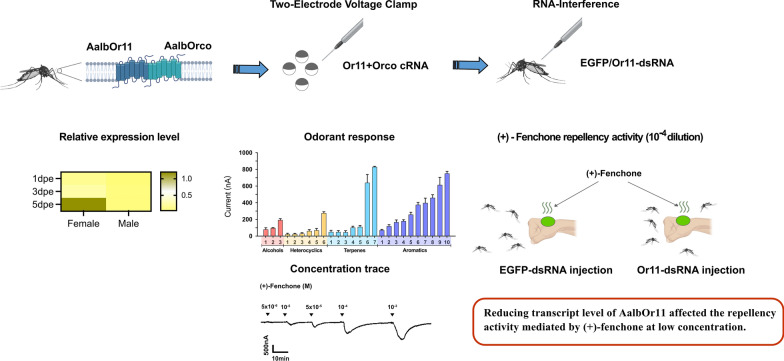

**Supplementary Information:**

The online version contains supplementary material available at 10.1186/s13071-022-05158-1.

## Background

Female *Aedes albopictus* is a vector for viral pathogens causing human diseases including yellow fever, dengue fever, and chikungunya. Due to strong ecological plasticity and a wide range of biting hosts, it is implicated in outbreaks of these diseases in areas where their primary vector, *Aedes aegypti*, is absent or outnumbered by *Ae. albopictus* [3, 6, 11, 15, 16, 35, 41]. These arboviral diseases carried by *Ae. albopictus* are increasingly becoming a global health concern [21, 29]. Many interventions against these vector-borne diseases have long relied on reducing mosquito populations. Besides insecticides, odor-baited traps which depend on the mosquito olfactory system are a major strategy [26, 31, 32]. Therefore, a better understanding of the relationships between the *Ae. albopictus* olfactory system and odorants might provide important information for developing active semiochemicals for monitoring or controlling populations.

The olfactory system is essential for mosquitoes, since vital behaviors such as finding carbohydrate sources, hosts for blood meals, and oviposition sites and avoiding predators are dependent primarily on its detection of blends of volatile molecules from the complex chemical world [12]. As two major olfactory organs, antennae and maxillary palps both contain many hair-like sensilla, which house olfactory sensory neurons (OSNs) for detecting odorants. In most instances, two (up to four) OSNs coexist in one sensillum, and each OSN expresses one odorant receptor protein [10]. The function of odorant receptors (Ors) requires co-expression with a highly conserved receptor (known as Orco). The Or-Orco complex that is formed is a ligand-gated heterodimeric cation channel that can open directly upon activation by an appropriate ligand [10, 20].

Mosquitoes possess various numbers of the Or family, varying from 18 (*Anopheles darlingi*) to 180 (*Culex quinquefasciatus*) [1, 2, 9, 18, 23]. As one model for studying olfactory chemosensing, all Ors of *Anopheles gambiae* have been identified and the majority has been deorphanized, which means that corresponding ligands of Ors have been found [42]. In comparison to *An. gambiae*, *Ae. albopictus* has extremely different behaviors, such as daytime biting and oviposition sites in containers [8, 14, 27, 36], which may explain the difference in their odorant reception to different chemical stimuli. However, only several Ors of *Aedes* have been defined; for example, AaegOr4 is considered to be the key receptor for distinguishing humans from other animals [25], and AalbOr2 and AalbOr10 respond strongly to indole and skatole from oviposition sites [22, 39]. Due to the low homology of odorant receptors among different insect species [6], most odorant receptors in *Aedes* are still "orphan receptors" (no corresponding ligands have been found), which hampers our understanding of its olfactory system.

According to previous RNA sequencing (RNA-seq) data, we found that non-blood-fed (NBF) female mosquitoes possessed higher transcript levels of AalbOr11 than males (unpublished). In addition, the alignment of amino acid sequences indicated that Or11 was conserved among three major disease-transmitting vectors: *Anopheles*, *Culex*, and *Aedes* (Additional file 1: Figure S1). Such conserved and female-biased odorant receptors as Or11 should be significant for the olfactory system in mosquitoes; thus, we deorphanized AalbOr11 in *Xenopus* oocytes by a panel of odorants with physiologically and behaviorally relevant compounds, including human-related odorants, oviposition attractants, and plant repellents [13, 28, 42]. The expression profiles of AalbOr11 among different sexes, ages, and physiological states were investigated. The expression and functional profiles suggested that AalbOr11 might be involved in mosquito host-seeking. Furthermore, we used a strong ligand of AalbOr11, (+)-fenchone, as the subject to explain the mechanism of odorant-induced behavior.

## Methods

### Mosquito strains

The colony of *Ae. albopictus* used in this study was kindly provided by Zhejiang Provincial Center for Disease Control and Prevention, China. The colony was originated from a population collected from Sichuan, China, and has been maintained in insectary for 15 years without exposure to any insecticides. Mosquitoes were maintained at 27 ± 1 °C, 70 ± 10% relative humidity (RH), with a photoperiod of 14:10 (light/dark).

### RNA isolation and cDNA synthesis

To assess the relative transcript abundance of AalbOr11 in different sexes and ages, gene expression assays were conducted using the antennae of male and non blood-fed female mosquitoes at 1, 3, and 5 days post-eclosion (dpe). In addition, the mosquitoes were collected at 1 h, 48 h, and 96 h after blood-feeding to identify the effects of the blood-feeding behavior on relative transcript abundance of AalbOr11. After the mosquitoes were cold-anesthetized, their antennae were cut off with dissecting scissors and immediately placed in RNAlater-ICE (Ambion, Austin, TX) on ice prior to RNA extraction. The total RNA was extracted using TRIzol reagent (TaKaRa, Tokyo, Japan) and isolated according to the manufacturer’s instructions. Then, 1 μg of total RNA was used as template for complementary DNA (cDNA) synthesis by reverse transcription using a PrimeScript™ RT Reagent Kit with gDNA Eraser (TaKaRa Bio, Otsu, Japan) following the manufacturer’s instructions.

### Quantitative analysis of transcription levels

The expression profile of AalbOr11 was determined using reverse transcriptase quantitative polymerase chain reaction (RT-qPCR). β-actin was used as the housekeeping gene (primer sequence: Actin-qF:GCTACGTCGCCCTGCACTT; Actin-qR: AGGAACGACGGCTGGAAGA), and qPCR was performed using a TB Green Premix Ex Taq II Kit (TaKaRa, Tokyo, Japan). Each 20 μL qPCR reaction mixture consisted of 10 μL 2× TB Green Premix Ex Taq II mix (Tli RNase H Plus), 0.8 μL of each primer (10 μM), 2 μL diluted cDNA template, 0.4 μL ROX Reference Dye II (50X) [36], and 6 μL sterilized deionized water. The primers were as follows: AalbOr11-qF: 5′-ATGCAGCTCAAAGACGAAT-3′; AalbOr11-qR: 5′-AGCAGAATCCATAGTACT -3′. The qPCR was conducted on a QuantStudio 3 Real-Time PCR Detection System (Applied Biosystems) under the following conditions: 95 °C for 30 s, followed by 40 cycles of 95 °C for 5 s, 60 °C for 34 s. The reproducibility was validated by including three technical replicates and three biological replicates for each reaction. Acquisitive data were analyzed with the 2^−ΔΔCt^ method.

### In vitro functional characterization of Ors

Gene-specific primers were designed based on coding sequences of putative AalbOr11 (XM_029861619.1) and identified AalbOrco (AALF000221/XM_029877254.1). PCR was performed using the following gene-specific primers containing Kozak motif (GCCACC): AalbOr11 F: GCCACCATGCAGCTCAAAGACGAATGGAT; AalbOr11R: TTAGCCGGCAGCTTGCTTCAGGA; AalbOrco F: GCCACCATGAACGTCCAGCCGACAAAGTA; AalbOrco R: TTATTTCAACTGCACCAACACCA. Subsequently, PCR products through sequencing validation were subcloned into pT7TS vector with the In-Fusion HD cloning kit (TaKaRa Bio, Otsu, Japan). The capped RNA (cRNA) was prepared from linearized vectors and purified by mMESSAGE mMACHINE T7 kit (Ambion, Austin, TX) according to the manufacturer’s instructions. Mature healthy oocytes (stage V–VII) were isolated from female *Xenopus laevis* frog ovarian lobes using standard procedures as described previously [4]. Oocytes treatment and purified cRNA microinjection were consistent with those described previously [42]. Each oocyte was injected with 27.6 nL of AalbOr11 and AalbOrco cRNA at a 1:1 ratio. Post-injection, oocytes were kept at 18 °C for 3–7 days in incubation buffer (1× Ringer’s solution supplemented with 5% dialyzed horse serum, 50 μg/mL tetracycline, 100 μg/ml streptomycin, and 550 μg/mL sodium pyruvate).

Odorant stock solutions were prepared at 10^–1^ M with DMSO. Odorant-induced current at a holding potential of –80 mV was recorded from the injected *Xenopus* oocytes using a two-electrode voltage-clamp setup (RC-3Z/OC-725D, Warner Instruments). Data acquisition and analysis were carried out with an Axon Digidata 1550B and pCLAMP 10 software (Molecular Devices, LLC, Sunnyvale, CA). Information regarding odorants used in electrophysiological recordings is provided in Additional file 2: Table S1.

### Double-stranded RNA (dsRNA) synthesis and microinjection

The genes of interest (GOI) were amplified using gene-specific primers that included T7 promoter sequences (underlined), AalbOr11-F: TAATACGACTCACTATAGGGACGACGTTTACGACAATCCG, AalbOr11-R: TAATACGACTCACTATAGGGATCCCAGAAAATCGCCTTCT; EGFP-F: TAATACGACTCACTATAGGGCCACAAGTTCAGCGTGTCCG, EGFP-R: TAATACG. ACTCACTATAGGGAAGTTCACCTTGATGCCGTTC. PCR products were amplified with Phusion High-Fidelity DNA Polymerase (NEB, Ipswich, MA), and the PCR procedure was conducted with the following settings: 98 °C for 30 s, 35 cycles of 98 °C for 15 s, 60 °C for 30 s, and 72 °C for 10 s, followed by a final 10 min extension step at 72 °C. The purified and sequenced PCR fragments were used as the template for synthesizing dsRNA of AalbO11 and EGFP. The dsRNA molecules were synthesized using the TranscriptAid T7 High Yield Transcription Kit (Ambion, Austin, TX) according to the manufacturer’s protocol.

Three-day-old female mosquitoes were collected and placed on a clean CO_2_ pad to be anesthetized. The anesthetized mosquitoes were lined up on the side for injection, and 1000 ng of dsRNAs in 0.5 μL volume was injected into one side of the thorax with a PLI-100 injector (World Precision Instruments). Post-injection, mosquitoes were put in new cages and supplied with sugar water (10% wt/vol). Males (ratio 1:1) were released into the cage for mating.

### Hand-in-cage assay

The hand-in-cage assay method used was identical to that described previously [44]. Forty 4- to 9-day-old females (mated, non blood-fed) were transferred to a 30 cm × 30 cm × 30 cm mosquito cage for 24 h prior to assay and were provided only with water in a cotton ball. Each compound dissolved in 500 µL acetone was evenly applied to a piece of nylon netting (mesh size 0.5 mm, 7 cm × 6 cm) and dried in air for 5 min. A window (6 cm × 5 cm) was cut out in a nitrile glove, and a set of magnetic window frames was assembled and put on a modified glove. The magnetic window frames contained one piece of magnetic frame, one treated net, three magnetic frames, one untreated net, and one magnetic frame from bottom to top. In this case, mosquitoes were attracted to skin emanations from the hand through the open window but were unable to contact treated nets with tarsi. The assay was performed at a temperature of around 28 °C and around 50% RH. The assay was video-recorded for 5 min, and the number landing on the test window and trying to pierce the skin was counted from the second to fifth minutes. For each cage, control (acetone) was tested before treatment, and the control with a high landing number was used to test compounds after 1 h to allow mosquitoes to fully recover and residual vapors from experiments to dissipate. Percentage repellency was determined using the following equation: Percentage repellency = [1 − (cumulative number of mosquitoes on the window of treatment from 2 to 5 min / cumulative number of mosquitoes on the window of solvent treatment for from 2 to 5 min)] × 100 [5].

## Results

### Transcript level of female-biased AalbOr11 decreased significantly after a blood meal

**Fig. 1:**
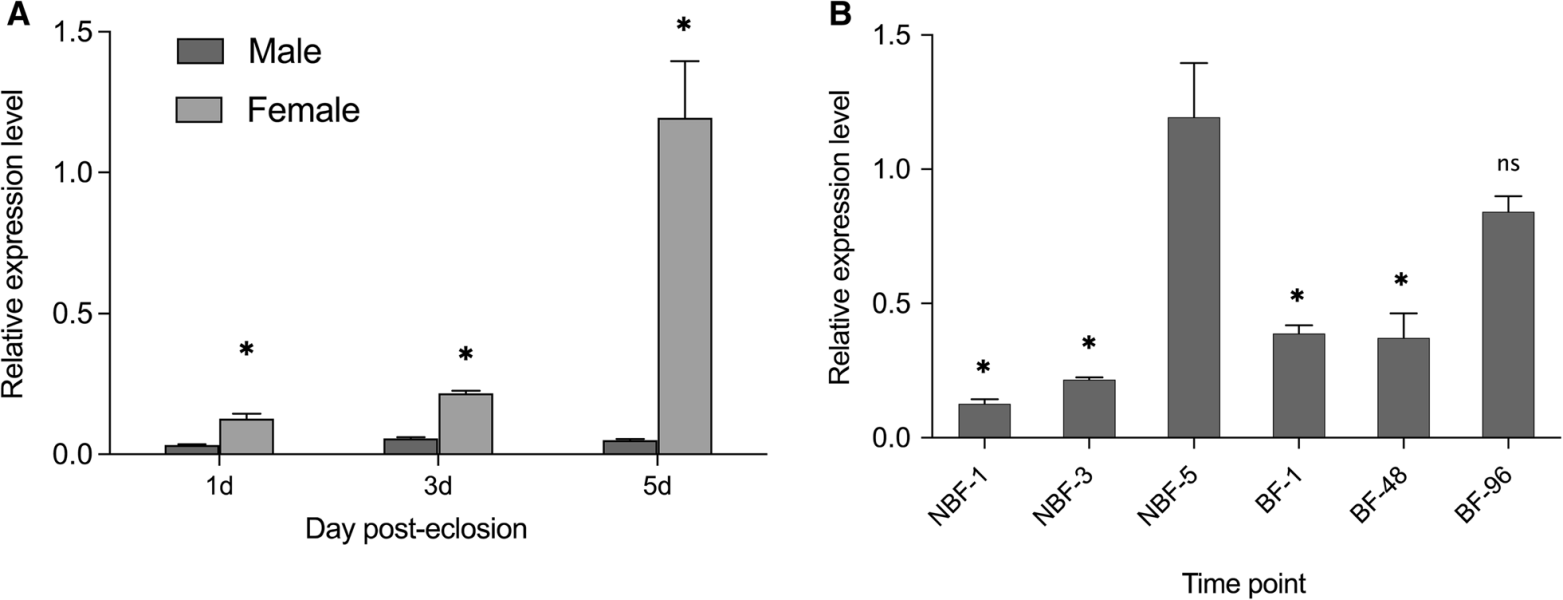
Transcript levels of AalbOr11 in *Ae. albopictus* antennae. **a** AalbOr11 transcript levels in antennae of male and female mosquitoes at 1, 3, and 5 day post-eclosion. **b** AalbOr11 transcript levels in antennae of non-blood-fed (NBF, 1, 3, and 5 days post-eclosion) and blood-fed (BF, 1 h, 48 h, and 96 h after blood-feeding) female mosquitoes. Data are plotted as mean ± SEM, *n* = 4. Statistical analysis was conducted using Student’s unpaired *t*-test (ns, not significant, *P* > 0.05; *, *P* < 0.05)

We performed RT-qPCR assay on *Ae. albopictus* to identify the transcript level of AalbOr11 in mosquitoes of different sexes, ages, and physiological states. The transcription level of AalbOr11 in females was significantly higher than that in males among 1 dpe (*P* = 0.0021), 3 dpe (*P* = 0.0000001), and 5 dpe (*P* = 0.00005) mosquitoes (Fig. 1a). The expression level of AalbOr11 in female mosquitoes exhibited a stark change between 1 and 5 dpe (*P* = 0.0059; Fig. 1b), as well as between 3 and 5 dpe (*P* = 0.0012; Fig. 1b). To further define the function of AalbOr11 in odor-induced behavior, we analyzed the dynamic changes in different physiological states in female mosquitoes, including non blood-fed mosquitoes at 1 dpe, 3 dpe, and 5 dpe (termed NBF-1, NBF-3, NBF-5) and blood-fed mosquitoes at 1 h, 48 h, and 96 h (termed BF-1, BF-48, BF-96). The transcript level of AalbOr11 in BF-1 or BF-48 mosquitoes was significantly lower than that in NBF-5 samples (*P*_BF-1_ = 0.0038; *P*_BF-48_ = 0.0033, Fig. 1b). However, there was no significant difference in AalbOr11 abundance between NBF and BF-96 mosquitoes (*P* = 0.1252, Fig. 1b).

### AalbOr11 appeared to be a wide-tuned receptor

**Fig. 2:**
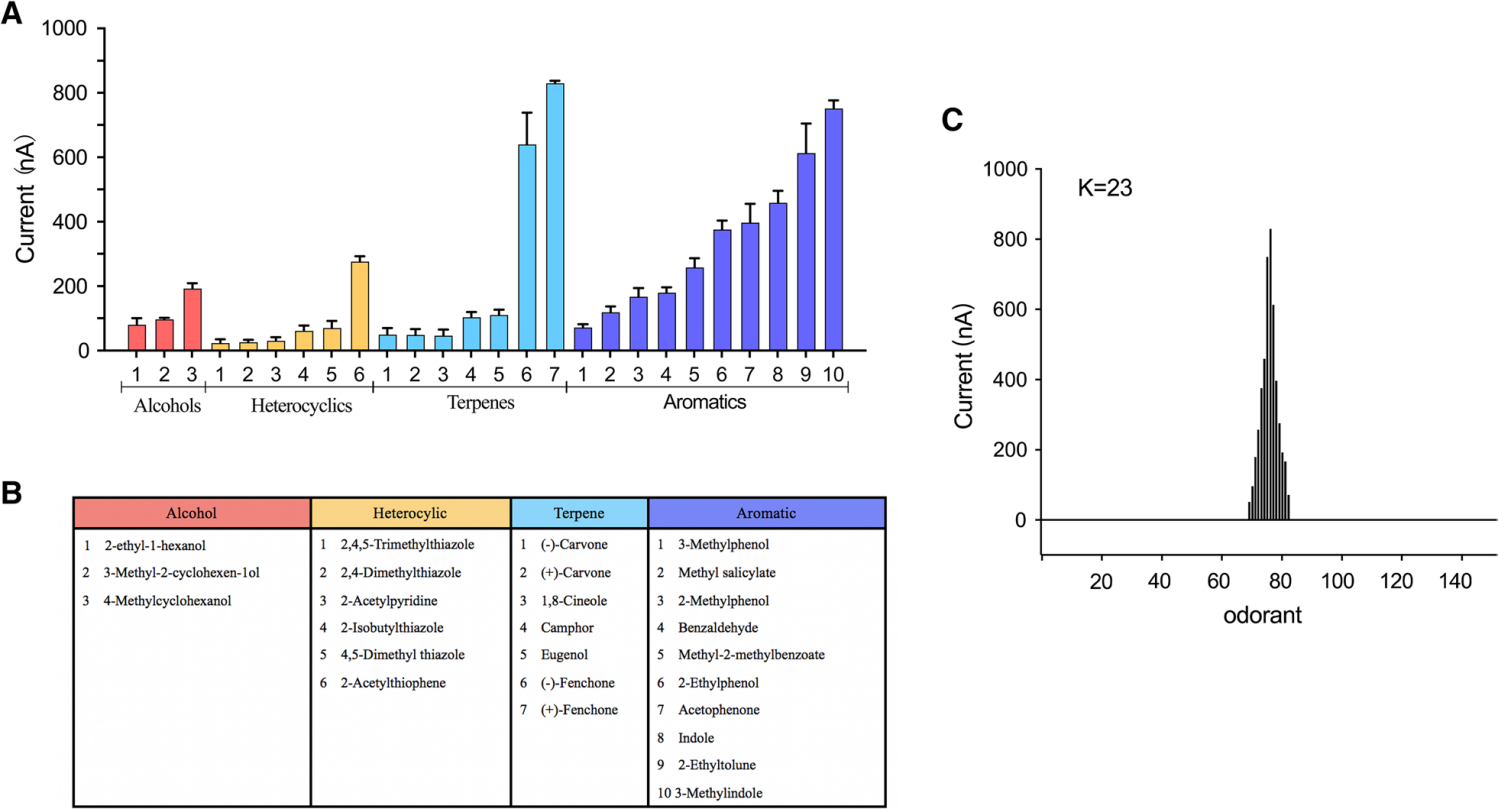
Current response and tuning curves of AalbOr11. **a** Current response recorded from oocytes expressing AalbOr11/AalbOrco (mean ± SEM, *n* = 8). The columns with different colors are classified into four catalogs according to chemical structure. **b** Each catalog displays the active compounds. **c** Tuning curves of AalbOr11. The 125 odorants are displayed along the *x*-axis, with those eliciting the strongest response placed near the center, and those eliciting the weaker responses placed near the edges. The kurtosis value is indicated in the graph

We co-expressed AalbOr11 along with AalbOrco in *Xenopus* oocytes for deorphanization. A panel of odorants including human-related odorants, oviposition attractants, and plant repellents was used to identify the ligands of AalbOr11. According to chemical structures, 126 odorants were classified into 11 major chemical categories: terpenes, alcohols, esters, aromatics, heterocyclics, acids, aldehydes, ketones, amines, lactones, and compounds from pyrethrum. A high dosage (10^–4^ M), was used as the preliminary screening concentration. A total of 26 odorants elicited currents on AalbOr11/AalbOrco, of which the currents induced by seven odorants were greater than 300 nA. The seven strong ligands comprised two terpenes (+)-fenchone and (−)-fenchone, and five aromatics 3-methylindole, 2-ethyltoluene, indole, acetophenone, and 2-ethylphenol (Fig. 2a, b). In order to identify whether AalbOr11 was a specialist or a generalist, Or tuning curves [17] were generated (Fig. 2c). As shown in Fig. 2c, AalbOr11 responded to 26 chemically diverse odorants, and could be classified as a generalist.

**Fig. 3:**
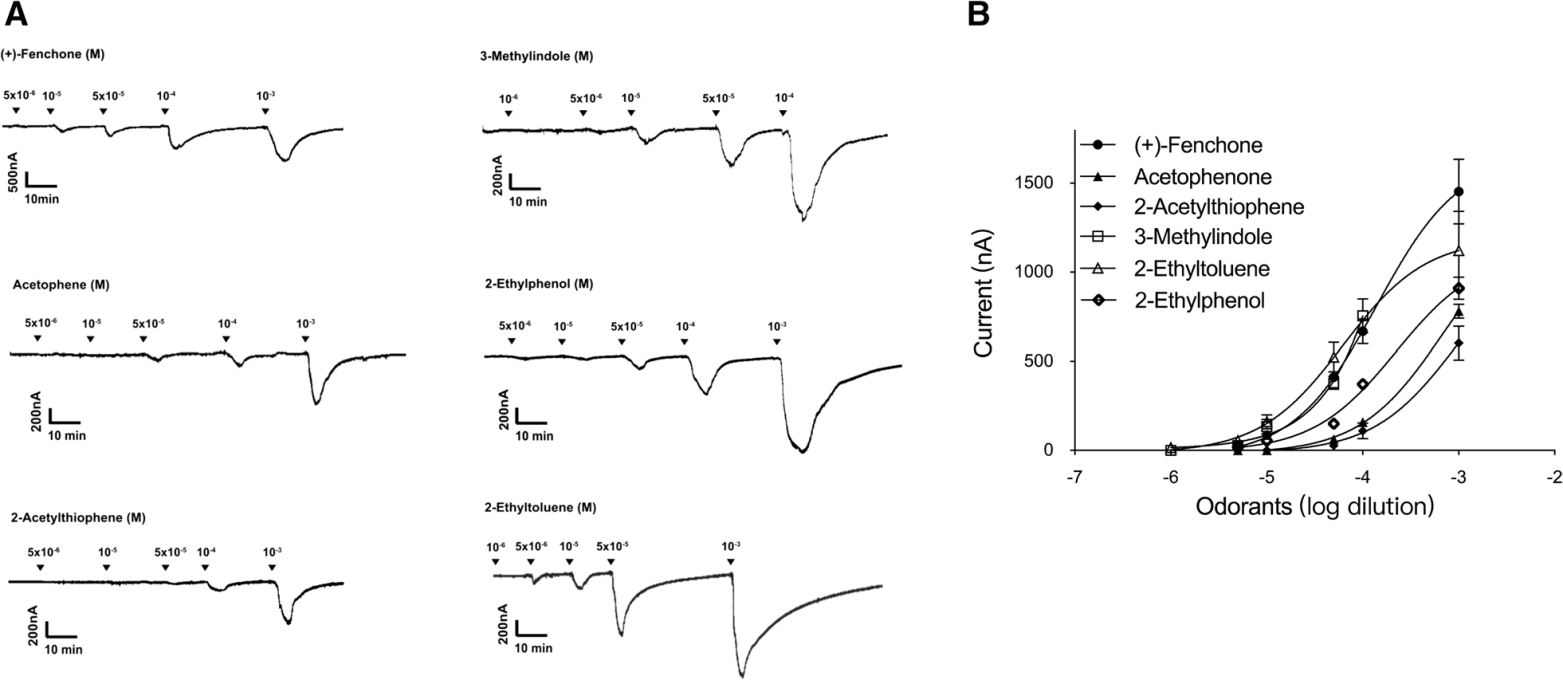
Concentration–response relationships of AalbOr11/AalbOrco to test compounds. **a** Traces obtained with a single oocyte challenged with a range of six ligand concentrations. **b** Concentration-dependent relationships between AalbOr11 and its strongest ligands. Mean ± SEM, *n* = 4–6 for each point. Data obtained with different oocytes were not normalized

Subsequently, we performed dose–response analyses for the best ligands to obtain more information about the sensitivity of the AalbOr11 receptor. In consideration of the similar structure of indole and 3-methylindole, indole was replaced with 2-acetylthiophene to conduct concentration gradient assay. Since these six ligands could elicit greater current at 10^–3^ M doses, we conducted concentration–response analyses in a range from 10^–3^ to 10^–6^ M. The strongest ligand, (+)-fenchone, elicited robust mean current (~ up to 1500 nA) at a dose of 10^–3^ M, whereas at a dose of 5 × 10^–6^ M, (+)-fenchone only induced current to 10 nA, which activated AalbOr11/AalbOrco at half-maximal effective concentration (EC_50_) of 139.5 μM. Another compound, 2-ethyltoluene, which belongs to the aromatics, elicited lower responses at a dose of 10^–3^ M; however, its EC_50_ was 62.19 μM, thus representing the most sensitive ligand (Fig. 3).

### Repellent activity of (+)-fenchone might be linked to odorant reception of AalbOr11

**Fig. 4:**
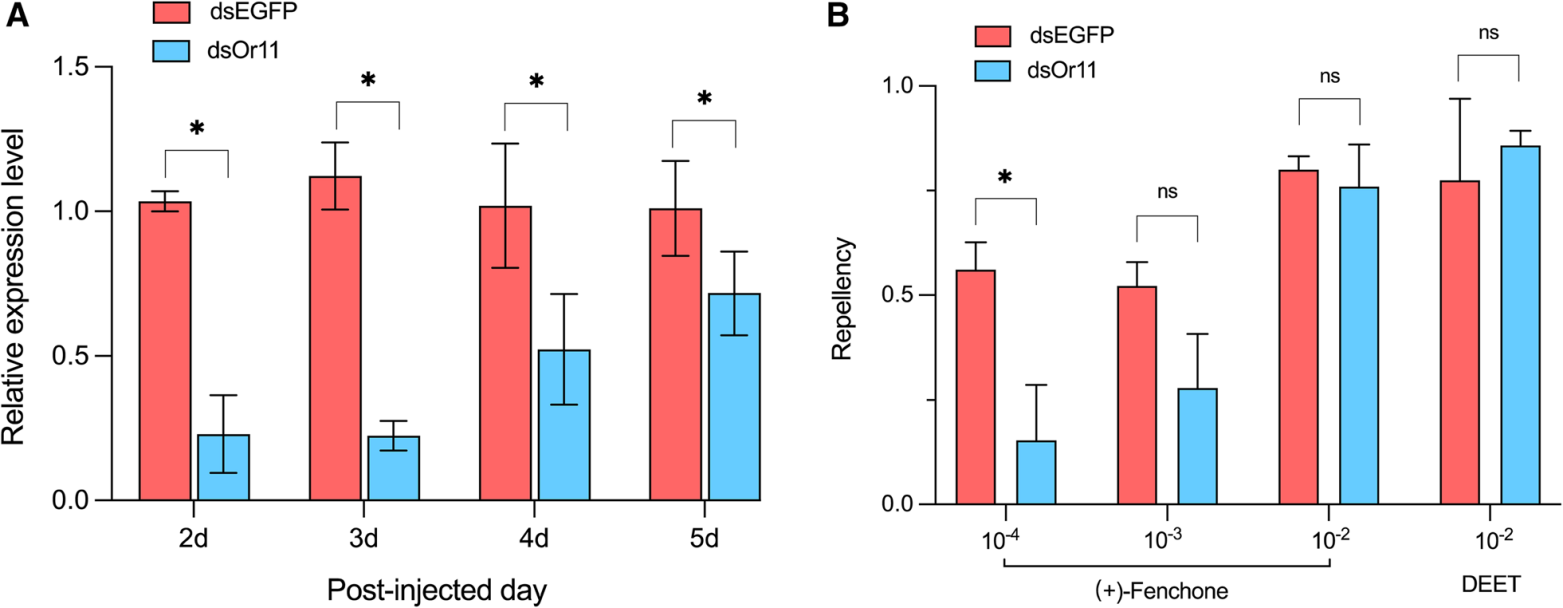
RNAi efficiency and hand-in-cage assays. **a** Transcript levels of AalbOr11 in 2-, 3-, 4-, and 5-day female mosquitoes after injection of AalbOr11-dsRNA and EGFP-dsRNA (mean ± SEM, *n* = 9). **b** Effect of AalbOr11 on the response of *Ae. albopictus* to (+)-fenchone and DEET (mean ± SEM, *n* = 8–12 cages). Statistical analysis was conducted using Student’s unpaired *t*-test (ns, not significant, *P* > 0.05; *, *P* < 0.05)

To further link odorant, behavior with the receptor, we used a strong ligand of AalbOr11, (+)-fenchone, as the subject to investigate odorant-induced behavior. Our hand-in-cage assay showed that (+)-fenchone elicited repellency in *Ae. albopictus* which was comparable to that of positive control DEET when at the 10^–2^ dilution (Fig. 4b). We conducted RNA interference (RNAi) experiments to assess whether reducing the transcript level of AalbOr11 would affect repellent activity. AalbOr11-dsRNA-treated mosquitoes had a lower abundance of AalbOr11 than mosquitoes injected with EGFP-dsRNA (*P* = 0.000012, Student’s unpaired two-tailed *t*-test), and the knockdown effect of AalbOr11 was maintained for at least 5 days (Fig. 4a). Then 2-day mosquitoes after dsRNA injection were used to compare repellent activity with hand-in-cage assay. We used three doses of (+)-fenchone (0.01%, 0.1%, 1%) to examine repellency, and used 1% doses of DEET as the positive control. At lower doses (0.01%), protection elicited by (+)-fenchone decreased significantly (*n* = 8, *P* = 0.0273) (Fig. 4b). Moreover, at a higher dose (0.1%), (+)-fenchone-induced protection was lower in AalbOr11-dsRNA-treated than in EGFP-dsRNA-treated mosquitoes, but the difference was not significant (*n* = 6–7, Student’s unpaired *t*-test, *P* = 0.1300) (Fig. 4b). By contrast, repellency elicited by the highest dose (1%) of (+)-fenchone was not significantly different between AalbOr11-dsRNA-treated and EGFP-dsRNA-treated mosquitoes, which was consistent with DEET repellency (*n* = 4, Student’s unpaired *t*-test, *P* > 0.999) (Fig. 4b).

## Discussion

Age-, sex-, and feeding state-dependent transcript level changes in odorant receptors have been found in other mosquitoes, such as *Ae. aegypti*, *Anopheles coluzzii*, and *An. gambiae* [33, 37, 40], which might result from their olfactory system modulation, so that mosquitoes can rapidly respond to chemical stimuli at the right moment. In this study, we found that female mosquitoes possessed higher transcript levels of AalbOr11 than males. Furthermore, the abundance of AalbOr11 in female mosquitoes reached its peak at 5 days post-emergence, and decreased significantly after blood-feeding. The phenomenon of blood meal-induced reduction in transcript levels of Ors has also been found in other mosquitoes, such as AgamOr46/47/48 [37] and AaegOr116 [24]. Our results indicate that AalbOr11 may be involved in blood-meal-seeking behavior. A previous study revealed that Or gene expression in *Ae. aegypti* antennae might contribute to human preference, and the differentially expressed Or4 responded to sulcatone, a human odorant, to discriminate human and non-human animals [25]. Our electrophysiological results also indicated that AalbOr11 was sensitive to human odor, such as indole, 3-methylindole, and methyl-2-methylbenzoate, which suggests that preference for human odor in mosquitoes is tightly linked to increases in expression.

In the ongoing evolution of mosquito species, Or11 is conserved among three major disease-transmitting vectors: *Anopheles*, *Culex*, and *Aedes*. AalbOr11 shared 58.39%, 58.49%, and 69.5% overall sequence identity with Or11 of *An. gambiae*, *An. coluzzii*, and *Cx. quinquefasciatus*, respectively. Or11 of *Ae. aegypti*, another mosquito species belonging to the subfamily *Aedes*, was up to 93.57% identical to AalbOr11 (Additional file 1: Figure S1). The high homology of Or11 among three major disease-transmitting mosquito genera suggests it may play a crucial role in many behavioral contexts. We wondered whether its odorant reception profile was consistent with OR11 of *An. gambiae* [42]. According to deorphanized results, we found that AalbOr11 was a broad-tuned receptor, which could be activated by ten aromatics, seven terpenes, six heterocyclics, and three alcohols. All ligands were consistent with AgOr11 except eugenol, which could activate AalbOr11 but could not activate AgOr11 [42]. Thus, Or11 homology was functionally conserved in different mosquito species, which was similar to that described previously in Or2 homology in *Ae. albopictus* [39], *Cx. quinquefasciatus* [34], and *An. gambiae* [7, 42]. In addition, the EC_50_ of the strongest ligand was 62.19 μM, far from nanomolar or picomolar concentration [38], suggesting that the truly strongest ligand of AalbOr11 was not found or that it is a broad-tuned but not sensitive receptor.

The strong ligand of AalbOr11, (+)-fenchone, has been found to confer repellency to several insects, including *Aedes aegypti* [19], *Drosophila melanogaster*, *Drosophila suzukii* [43], and *Prostephanus truncates* [30]. Similarly, repellent activity of (+)-fenchone against *Ae. albopictus* was found in this study. Hence we performed RNAi experiments to attempt to link odorant reception with repellent activity. The results showed that reducing the transcript level of AalbOr11 affected the repellent activity mediated by a lower concentration of (+)-fenchone (0.01% v/v). In addition, reduced protection was observed with knockdown mosquitoes at a higher concentration (0.1%), although it was not statistically significant. On one hand, (+)-fenchone-elicited repellency might involve multiple Ors of *Ae. albopictus*. On the other hand, RNAi treatment reduced transcript levels by only c. 80%, and the remaining AalbOr11 still played a significant role in (+)-fenchone-mediated repellent activity. The possible link between reception and behavior has also been found in *Cx. quinquefasciatus* [45]. The significant reduction in protection in CquiOr4-dsRNA-treated mosquitoes suggests it may play a significant part in 2-phenylethanol-mediated repellent activity [45]. RNAi and bioassay suggested that AablOr11 may be one of the receptors mediating (+)-fenchone repellency.

## Conclusion

We found that AalbOr11 was highly conserved among three major disease-transmitting vectors and was enriched in non blood-fed female mosquitoes. According to in vitro functional characterization, AalbOr11 was a broadly tuned receptor. In addition, RNAi and bioassay suggested that AablOr11 may be one of the receptors mediating (+)-fenchone repellent activity. Our study provides further information regarding the mechanisms of olfactory-mediated mosquito behavior (e.g., host-seeking and repellent activity), and also provides new insight into active semiochemical identification for monitoring or controlling mosquito populations.

## Supplementary Information

**Additional file 1: Figure S1.** Aligned amino acid sequence from *Aedes*, *Culex*, and *Anopheles*. AablOr11 refers as *Aedes albopictus* Or11, AaegOr11 refers to *Aedes aegypti* Or11, CquiOr11 refers to *Culex quinquefasciatus* Or11, CpipOr11 refers to *Culex pipiens pallens* Or11, AgamOr11 refers to *Anopheles gambiae* Or11, AcolOr11 refers to *Anopheles coluzzii* Or11.

**Additional file 2: Table S1.** Compounds used in both electrophysiological recordings and behavioral assays.

## Data Availability

All data generated in this study are presented within this published article.

## Abbreviations

RT-qPCR: Reverse transcriptase quantitative polymerase chain reaction
RNAi: RNA interference
Or: Odorant receptor
Orco: Odorant co-receptor
OSN: Olfactory sensory neuron
cDNA: Complementary DNA
cRNA: Capped RNA
dsRNA: Double-stranded RNA
dpe: Days post-eclosion
DEET: *N*,*N*-Diethyl-3-methylbenzamide
EC_50_: Half-maximal effective concentration
NBF: Non blood-fed
BF: Blood-fed
RNA-seq: RNA sequencing
