# Supplementary material for: Molecular and functional characterization of a conserved odorant receptor from Aedes albopictus
Source: Parasit Vectors. 2022 Jan 31;15:43. doi: 10.1186/s13071-022-05158-1 (PMC8805257; doi:10.1186/s13071-022-05158-1)
Supplement: Supplementary file 2 — Additional file 2: Table S1. Compounds used in both electrophysiological recordings and behavioral assays. [file 13071_2022_5158_MOESM2_ESM.pdf]

Table S1. Compound used in both electrophysiological recordings and behavioral assays.

Chemical name	CAS	Purity	Source
geraniol	106-24-1	>96.0%	TCI
Linolooloxide	60047-17-8	>97.0%	TCI
(-)-carvone	6485-40-1	99.00%	TCI
(-)-fenchone	7787-20-4	98.00%	TCI
(+)-Carvone	2244–16-8	99.00%	Supelco
(+)-fenchone	4695-62-9	99.50%	Supelco
citronellal	106-23-0	>98.0%	TCI
geranyl acetate	105-87-3	≥97%	Sigma
limonene	138-86-3	98.00%	TCI
citral	5392-40-5	>95.0%	Sigma
myrcene	123-35-3	98.00%	TCI
1,8-cineole	470-82-6	>96%	TCI
alpha-Terpinene	99-86-5	>95.0%	Sigma
p-Cymene	99-87-6	99.00%	Sigma
Camphor	76-22-2	>90%	TCI
a-pinene	80-56-8	98.00%	Sigma
2,3-butanedione	431-03-8	>98.0%	TCI
2-butanone	78-93-3	≥99.0%	Sigma
2-heptanone	110-43-0	99.00%	TCI
2-Nonanone	821-55-6	≥99%	Sigma
3-Octanone	106-68-3	≥99%	TCI
6-Methyl-5-hepten-2-one	110-93-0	98.50%	TCI
acetone	67-64-1	≥99.5%	Sigma
Cyclohexanone	108-94-1	>98.0%	TCI
1-butanol	71-36-3	>96.0%	TCI
1-Dodecanol	112-53-8	>99.0%	TCI
1-hepten-3-ol	4938-52-7	>98.0%	TCI
1-hexanol	111-27-3	>98.0%	TCI
1-Hexen-3-ol	4798-44-1	>98.0%	TCI
1-octen-3-ol	3391-86-4	≥98%	Sigma
1-pentanol	71-41-0	>95.0%	TCI
2-ethyl-1-hexanol	104-76-7	98.50%	TCI
3-Methyl-1-butanol	123-51-3	>99.5%	TCI
3-Methyl-2-cyclohexen-1ol	21378-21-2	96.00%	Sigma
4-Methylcyclohexanol	589-91-3	≥99.0%	Sigma
cis-3-hexen-1-ol	928-96-1	≥98%	Sigma
ethanol	64-17-5	≥99.0%	aladdin
methanol	67-56-1	>99.8%	TCI
Z2-hexenol	928-94-9	95.00%	Sigma
1-Octanol	111–87-5	>95.0%	TCI
Cyclohexanol	108–93-0	>99.0%	TCI
citronellol	7540-51-4	>90.0%	TCI
1-octyn-3-ol	818-72-4	98.00%	Sigma

linalool	78-70-6	>96.0%	TCI
1-Chlorododecane	112-52-7	97.00%	TCI
2,4,5-Trimethylthiazole	13623-11-5	>99.8%	TCI
2-Acetylpyridine	1122-62-9	98.00%	TCI
2-Acetylthiazole	24295-03-2	≥99%	Sigma
2-acetylthiophene	88-15-3	95.00%	Sigma
2-Ethoxythiazole	15679-19-3	>99%	TCI
2-Isobutylthiazole	18640-74-9	97.00%	TCI
4,5-dimethyl thiazole	3581-91-7	≥99%	Sigma
4-methylthiazole	693-95-8	>93%	TCI
Thiazole	288-47-1	99.00%	Sigma
2,4-Dimethylthiazole	541–58-2	>99%	TCI
2-Ethylphenol	90-00-6	>98.0%	TCI
2-Methylphenol	95-48-7	>99.0%	TCI
2-Ethyltoluene	611-14-3	≥98%	Sigma
2-Phenoxy ethanol	122-99-6	98.00%	TCI
2-propylphenol	644-35-9	97.00%	TCI
3-Methylphenol	108-39-4	>98.0%	TCI
3-methylindole	83-34-1	>98.0%	TCI
4-ethylphenol	123-07-9	>97.0%	TCI
4-Methylphenol	106-44-5	>99.0%	TCI
Acetophenone	98-86-2	>98.5%	TCI
benzaldehyde	100-52-7	>98.0%	TCI
benzyl acetate	140-11-4	97.00%	TCI
indole	120-72-9	>99.0%	TCI
Methyl benzoate	93-58-3	>99.0%	TCI
methyl salicylate	119-36-8	>99.0%	TCI
Phenethyl acetate	103-45-7	>99.0%	TCI
phenol	108-95-2	>99.5%	TCI
4-Propyl benzaldehyde	28785–06-0	95.00%	Sigma
pentanoic acid	109-52-4	>98.0%	TCI
Heptanoic acid	111-14-8	>98.0%	TCI
Nonanoic acid	112-05-0	98.50%	TCI
octanoic acid	124-07-2	≥98%	Sigma
Dodecanoic acid	143-07-7	98.50%	TCI
7-octenoic acid	18719-24-9	>99.0%	TCI
Decanoic acid	334-48-5	≥99%	Sigma
octadecanoic acid	57-11-4	>98.0%	TCI
Tridecanoic acid	638-53-9	>99.0%	TCI
isobutyric acid	79-31-2	>99.0%	TCI
hexanoic acid	142-62-1	≥99%	Sigma
propanoic acid	79-09-4	>99.0%	TCI
oleic acid	112–80-1	98.00%	TCI
Undecanoic acid	112–37-8	>98.0%	TCI
cadaverine	462-94-2	99.00%	Supelco
putrescine	110-60-1	97.00%	TCI

amyl acetate	628-63-7	>99.0%	TCI
ethyl acetate	141-78-6	>98.0%	TCI
ethyl butyrate	105-54-4	98.50%	TCI
Ethyl propanoate	105-37-3	98.00%	TCI
Isoamyl acetate	123-92-2	98.50%	TCI
isobutyl acetate	110-19-0	97.00%	TCI
Methyl caprylate	111-11-5	98.50%	TCI
methyl propanoate	554-12-1	>95.0%	TCI
propyl acetate	109-60-4	98.50%	TCI
Ethyl caproate	123–66-0	98.00%	TCI
trans-11-Octadecenoic Acid Methyl Ester	6198-58-9	>98.0%	TCI
cis-11-Octadecenoic Acid Methyl Ester	1937-63-9	98.50%	TCI
hexadecanoic acid methyl ester	112-39-0	99.50%	TCI
Ethyl oleate	111-62-6	>98.0%	TCI
ethyl acrylate	140-88-5	99.00%	TCI
cis-9-Hexadecenoic Acid Methyl Ester	1120-25-8	97.00%	TCI
Methyl butyrate	623-42-7	>99.5%	TCI
Decanal	112-31-2	98.00%	TCI
E2-hexenal	6728-26-3	98.00%	Sigma
Hexanal	66-25-1	>90%	TCI
Nonanal	124-19-6	99.00%	TCI
Octanal	124-13-0	98.00%	TCI
Δ-Decanolactone	705-86-2	>96%	TCI
gamma-decalactone	706-14-9	>98%	TCI
5-alpha-androst-16-one	18339-16-7	98.00%	TCI
Eugenol	97–53-0	>99.0%	TCI
thymol	89-83-8	>99.0%	TCI
EBF	18794-84-8	98.50%	Sigma
Pyrethrin I	121-21-1	≧99%	Purified
Pyrethrin II	121-29-9	≧99%	Purified
Cinerin I	97-12-1	≧99%	Purified
Cinerin II	204-454-2	≧99%	Purified
Jasmolin I	4466-14-2	≧99%	Purified
Jasmolin II	1172-63-0	≧99%	Purified
4-ketoisophorone	1125-21-9	>98.0%	TCI
4′-Ethylacetophenone	937-30- 4	97.00%	Sigma
DEET	134-62-3	99%	Sigma
